# Sequence-based comparison of field and vaccine strains of infectious bursal disease virus in Ethiopia reveals an amino acid mismatch in the immunodominant VP2 protein

**DOI:** 10.1007/s00705-020-04622-6

**Published:** 2020-04-13

**Authors:** Dereje Shegu, Teshale Sori, Asaminew Tesfaye, Alebachew Belay, Hawa Mohammed, Teferi Degefa, Belayneh Getachew, Takele Abayneh, Esayas Gelaye

**Affiliations:** 1 National Animal Health Diagnostic and Investigation Center, P.O. Box 04, Sebeta, Ethiopia; 2 Department of Clinical Studies, Addis Ababa University College of Veterinary Medicine and Agriculture, P.O. Box 34, Bishoftu, Ethiopia; 3 National Veterinary Institute, P.O. Box 19, Bishoftu, Ethiopia

## Abstract

Sequencing of the VP2 region was carried out to identify amino acid mismatches between vaccine strains and field isolates of infectious bursal disease virus (IBDV). Viruses were isolated in chicken embryo fibroblast (DF-1) cells using pooled samples of bursa collected from nine outbreaks, which affected 30,250 chickens in five localities, with an overall mortality of 47.87%. Virus strains were identified by comparing the deduced amino acid sequence between positions 232 and 446 of the immunodominant VP2 epitope. All of the pooled samples were positive for IBDV. RT-PCR yielded a 645-bp DNA fragment of the VP2 gene. Phylogenetic analysis of this fragment revealed clustering of these isolates with very virulent IBDV strains. The amino acid sequences of these isolates were identical to those of the European very virulent strains UK 661 and DV 86, except at position 222, but differed from the vaccine strains used in Ethiopia, suggesting the possible introduction of virulent virus strains to Ethiopia from Europe. Our study demonstrates the widespread presence of very virulent strains of IBDV on poultry farms in Ethiopia and demonstrates the need to evaluate the protective level of existing vaccines against circulating field viruses.

## Introduction

Chicken production plays an important role in Ethiopia, providing cash for women and children to buy clothing and school supplies, cover medical costs, and much more [8, 22]. Optimal utilization of chicken resources, however, is impaired by the considerable losses inflicted by infectious diseases such as infectious bursal disease (IBD). IBD is an acute, highly contagious viral disease of 3- to 6-week-old chickens that causes significant losses to the poultry industry worldwide [16]. The disease is caused by infectious bursal disease virus (IBDV), of which there are two known serotypes, designated serotypes 1 and 2 although clinical disease has been associated with only serotype 1 [30]. IBDV is spread through contaminated faeces, water and feed.

In Ethiopia, IBD was first reported in Debre Zeit in 2002 on a commercial farm where a live vaccine imported from the Netherlands was being used [32]. Since then, it has been prevalent in various regions [33], causing high mortality, ranging from 50% to 72%, in chickens [29, 34]. The disease is considered one of the important constraints to the poultry industry throughout the country [17].

Vaccination of chickens with both live attenuated and inactivated (killed) vaccines has been used to control the disease [30]. Vaccination of chickens against IBD has been implemented throughout Ethiopia since it was first reported. However, the vaccines used are either imported from abroad or produced locally from an imported master seed that has not been matched to the locally circulating field isolates. Vaccines produced using foreign infectious bursal disease virus (IBDV) strains may not be similar to field strains with respect to the amino acid sequence of the dominant epitope [31], and this could potentially result in vaccination failure. The frequent occurrence of outbreaks of IBD in several parts of the country despite vaccination suggests that there might be a mismatch between vaccines and field strains. The use of reverse transcriptase polymerase chain reaction (RT-PCR) to amplify the VP2 gene from IBDV has been used in other countries to identify vaccine mismatches [8]. Amino acid mutations at specific locations in hypervariable regions of the VP2 capsid protein are known to contribute to antigenic drift [10]. Hence, matching of vaccine strains with field isolates of IBDV based on their VP2 sequences can provide important information for ensuring the efficacy of vaccination. Such information is currently unavailable in Ethiopia. Here, we report the occurrence of a mismatch between commonly used vaccines and virulent field strains of IBDV in the amino acid sequence of the VP2 protein.

## Materials and methods

### Study areas

**Fig. 1:**
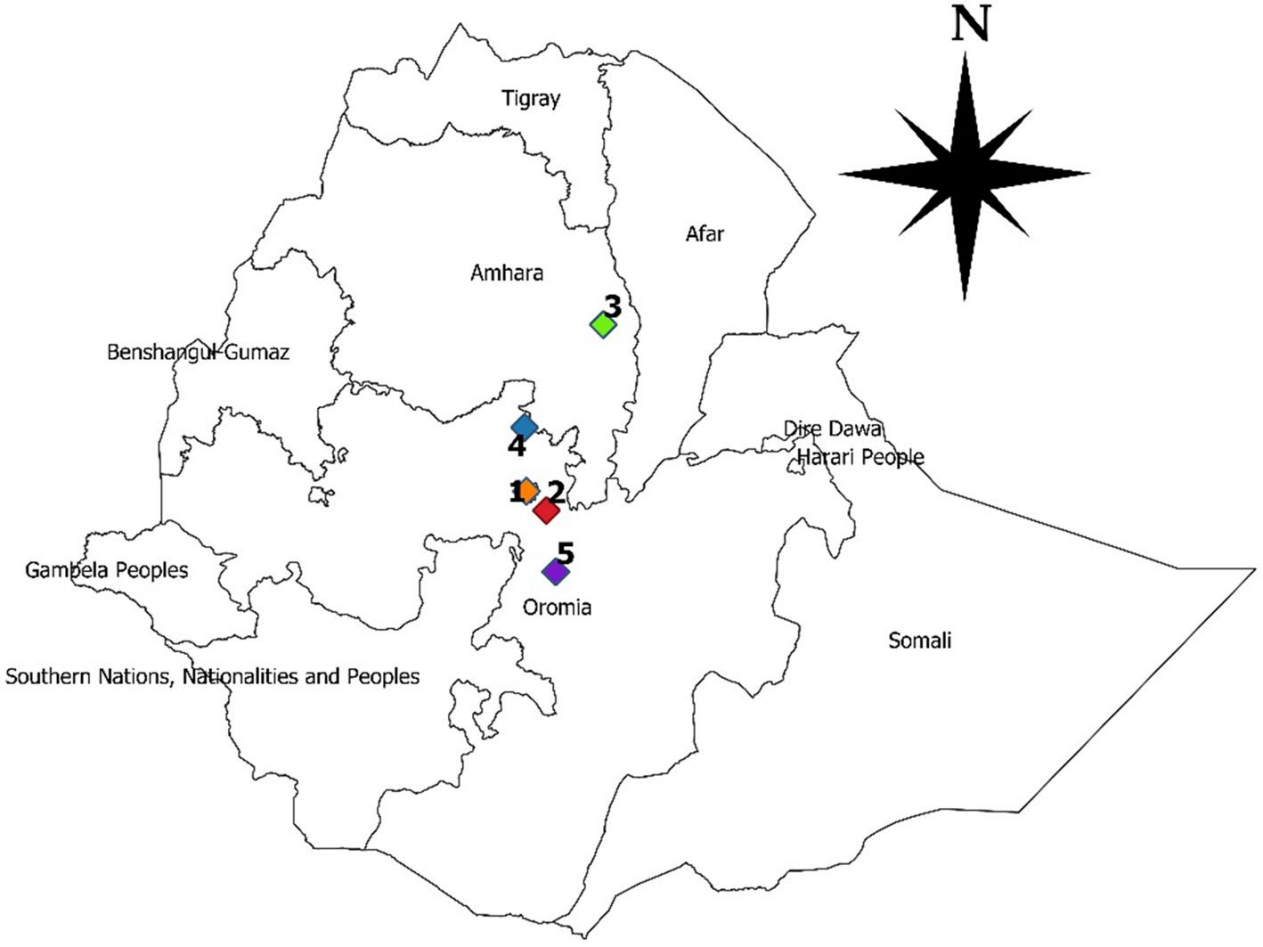
Map of Ethiopia showing the geographical locations where samples from suspected IBD outbreaks were collected and analyzed. 1, Addis Ababa; 2, Bishoftu; 3, Kombolcha; 4, Fiche/Sululta; and 5, Assela

The study was conducted on samples collected from five different areas of Ethiopia, namely, Addis Ababa, Bishoftu, Kombolcha, Fitche/Sululta and Assela (Fig. 1).

Addis Ababa is the capital city of Ethiopia, with an average elevation of 2300 meters above sea level (masl) and a subtropical climate. The average annual rainfall and average maximum and minimum temperature of Addis Ababa are 1180 mm, and 22.8 °C and 10.6 °C, respectively [4].

Bishoftu is a town close to Nazareth (as depicted on the map) where several commercial poultry farms of different scale, hatcheries, and breeding centers are found. Chickens of various ages and breeds are distributed from there to different parts of the country. It is located 47 km southeast of the capital, Addis Ababa, at 9 ºN latitude and 40ºE longitude, with an average altitude of 1850 meters above sea level and annual rainfall of 866 mm [21].

Kombolcha is located at 11º07″N latitude, 39º44″E longitude with an average altitude of 1864 masl. The area receives an annual rainfall of about 1038 mm and has an average temperature of 18 °C [3]. There is a government-owned commercial poultry farm center in Kombolcha from which farmers in the northeast parts of the country obtain chickens.

Fitche/Sululta is situated 40 km from Addis Ababa. Geographically it is located at 9º11′0″N latitude and 38º45′0″E longitude. The average elevation of the area is 2500 masl. The average annual temperature of the district is 14.7 °C with an average rainfall of 1119 mm [25]. The area is known for its commercial livestock production, especially dairy and poultry.

Assela is a town located in Arsi administrative zone, in the central part of the Oromia Regional State. It lies between 60º45″N and 80º58″N latitude and 38º032″E and 40º050″E longitude. It has a total area of 23881 km^2^ [4]. Livestock production, including poultry production, is an important economic activity in the area.

### Study population and sample collection and preparation

The study population consisted of broilers and layers raised on commercial poultry farms in the study areas affected by outbreaks of IBD. The farms investigated in the study areas used intensive husbandry practices. Sick birds and bursal tissues collected from suspected cases of IBD were submitted to the National Veterinary Institute (NVI) between June 2016 and February 2018 for isolation and identification of IBDV. The bursa samples were collected under aseptic conditions and transferred to sterile labeled universal bottles. The samples were transported to the research and development laboratories of NVI, maintaining the cold-chain using an icebox. In the laboratory, the samples were either processed immediately or kept at -80 °C until further processing [30].

The bursa samples were pooled together, with one pool consisting of five bursal tissues from five chickens from the same flock showing clinical signs of IBD. A total of 45 pools of bursal samples were collected and processed for virus isolation. The pools of bursa were chopped into small pieces using a sterile scalpel blade and minced using a mortar and pestle in a class II biosafety cabinet. A 10% (w/v) suspension of the bursal tissue was prepared in sterile phosphate-buffered saline containing penicillin (1000 units/mL) and streptomycin (1000 µg/mL). To facilitate the release of viruses from the tissues, the suspensions were freeze-thawed three times and centrifuged at 3000 *g* for 10 minutes [30]. The supernatant was harvested, filtered using a 0.22-μM Millipore filter, and stored at -20 °C before inoculation.

### Isolation of IBDV

The bursal suspensions were thawed, and 0.5 mL of the suspension was inoculated onto confluent chicken embryo fibroblast cells (DF-1, passage 10, obtained from GD, The Netherlands) in 25-cm^2^ tissue culture flask. After adsorption for 60 minutes at 37 °C, 10 mL of maintenance medium (Dulbecco’s modified Eagle’s medium [Sigma-Aldrich] supplemented with calf serum [Gibco] was added to each flask and incubated at 39 °C in a humidified incubator with 5% CO_2_. One uninoculated flask served as negative control. The cells were monitored daily for the development of a virus-induced cytopathic effect (CPE), using an inverted microscope. On the sixth day, the cultures were freeze-thawed, and the resulting lysate was inoculated onto fresh embryo fibroblast cells, and the procedure was repeated one more time [30].

### Molecular characterization of the isolates

#### RNA extraction and reverse transcription

Viral RNA was extracted from a suspension harvested from positive cultures using an RNeasy^®^ Mini Kit (QIAGEN, Austin, Texas, USA), following the manufacturer’s instructions. Reverse transcription was performed using a SCRIPTUM First strand cDNA synthesis kit (Bio and Sell Company). Amplification of the cDNA was performed targeting a partial sequence of the VP2 gene of IBDV using the gene-specific primers IBDF3 (5’-GTAAAACGACGGCCAGTGCATGCGGTATGTGACGCTTGGTCAC-3’) and IBDR3 (5’-CAGGAAACAGCTATGACCGAATTCGATCCTGTTGCCACTCTTTC-3’) [15, 30]. Throughout the amplification process, negative and positive controls were included.

The PCR reaction was performed in a final volume of 50 μL consisting of 20 µL of iQ™ Supermix (Bio-Rad), 16 µL of RNase-free water, 4 μL of forward primer, 4 µL of reverse primer, and 6 µL of cDNA template. The PCR reactions consisted of an initial 15 cycles of denaturation at 95 °C for 30 seconds, annealing at 60 °C for 30 seconds, and elongation at 72 °C for 30 seconds [15]. This was followed by 20 cycles of denaturation at 95 °C for 30 seconds, annealing at 56 °C for 30 seconds, and elongation at 72 °C for 30 seconds, with a final elongation step of 72 °C for 7 minutes. The amplified PCR products were visualized using 1.5% agarose gel electrophoresis in 1x TAE buffer. A 100-bp DNA ladder (Fermentas) was used, and IBDV-positive samples yielded a PCR product of 645 bp.

### Sequencing and phylogenetic analysis

The PCR products were purified using a Wizard SV Gel and PCR Clean-Up System kit (Promega, Germany), following the manufacturer’s instructions. The concentration of the purified PCR product was measured using a NanoDrop 2000c spectrophotometer (Thermo Scientific, USA). The purified PCR products were mixed with the sequencing primers and submitted to a commercial sequencing company (LGC Genomics, Berlin, Germany). The sequences from current isolates were assembled and edited using Vector NTI Advances™ 10 software (Invitrogen, USA). For comparative multiple sequence analysis, homologous sequences were retrieved from the GenBank database, and the sequences were aligned using BioEdit version 7.1.3.0 [9]. A multiple sequence alignment was made using ClustalW, and a phylogenetic tree was constructed using the neighbor-joining method with the maximum composite likelihood nucleotide substitution model. Pairwise comparisons were done using MEGA version 6 [26].

In addition to the isolates from this study, the commercially available vaccine strains LC75 (AviPro^®^ Precise, accession number EF429252), D78 (CLONEVAC D-78^®^, accession numbers AJ586963 and EU162087) and IBDL (CEVAC^®^ IBDL) as well as prototype very virulent strains (UK661 and DV86), classical virulent strains (Edgar and F52/70) and antigenic variants (DelE and GLS) were used for sequence comparisons.

## Results

### Isolation and identification of infectious bursal disease virus

**Fig. 2:**
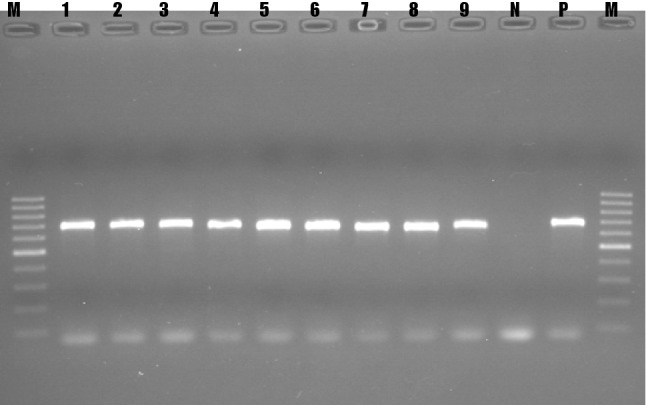
Gel electrophoresis of RT-PCR products from positive samples. M, DNA ladder (100 bp, Fermentas); lanes 1-9, samples from field outbreaks; N, negative control; P, positive control (645 bp)

Nine outbreaks affecting 30,250 chickens from five different poultry farms were investigated (Table 1). The largest number of cases was observed in Bishoftu, followed by Assela. Mortality ranging from 10% in Assela to 80% in Sululta was recorded, with an overall mortality of 48%. All of the pooled samples that were collected and cultured produced CPE, which was characterized by the appearance of small, round and reflective cells that were detached from the wall of the cell culture flask and floating in the medium. Nine of the samples that produced CPE were selected and tested using RT-PCR targeting the VP2 gene of IBDV, which resulted in a 645-bp PCR product (Fig. 2), confirming the identity of the virus.

**Table 1:** Mortality of chickens due to infectious bursal disease outbreaks in five localities observed in this study

Site	No. affected	Mortality (%)
Bishoftu	10700	66
Addis Ababa	3200	42
Assela	7350	10
Kombolcha	5000	41
Fitche/Sululta	4000	80
**Total**	**30250**	**48**

### Results of sequencing and phylogenetic analysis

**Fig. 3:**
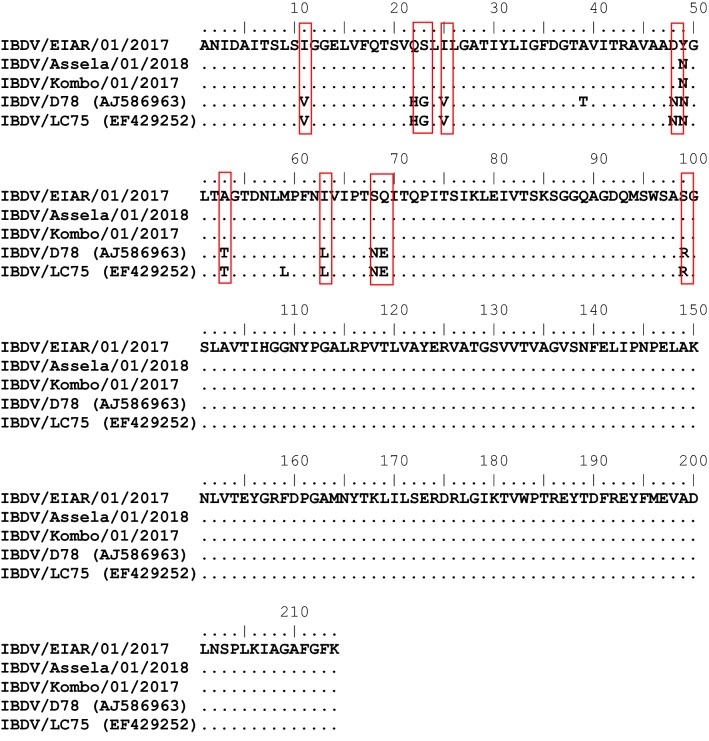
Comparison of amino acid sequences of the immune dominant VP2 variable domain (aa 232-446) of field isolates of IBDV and vaccine strains

The results of sequencing after editing of the raw sequence data and aligning the deduced amino acid sequences from positions 232 to 446 showed that the IBDV isolates from the outbreaks investigated in this study were all very virulent IBDV strains (Table 2; Fig. 3). The IBDV isolates from Bishoftu (designated as IBDV_EIAR), Asella (designated as IBDV_MB267), and Kombolcha (designated as IBDV_MB38) had amino acid sequences that were more similar to those of the British strain UK661 and the Dutch strain DV86 than to the vaccine strains commonly used to vaccinate chickens in Ethiopia, such as LC75 and D78. The isolates from this study differed from the European strains only at position 222, where the Ethiopian isolates had a gap. Sequences of the current isolates were identical to those of strains from Ethiopia that were characterized previously and whose sequences were deposited in the GenBank database. The VP2 gene nucleotide sequences of the isolates from this study were deposited in the GenBank database with the following accession numbers: IBDV_EIAR_Bishoftu_2016, MN422352; IBDV_Kombolcha_2017, MN422353; IBDV_Assela_2018, MN422354.

**Table 2:** Deduced amino acid differences at aa positions 232-446 between field isolates of IBDV, vaccine strains and prototype strains of vv, cv and av IBD viruses

Strain	Molecular type	Key amino acids position				
		222	242	256	294	299
EIAR	VV	-	I	I	I	S
Assela	VV	-	I	I	I	S
Kombolcha	VV	-	I	I	I	S
CEVACIBDL	CV	P	V	V	I	N
D78	CV	P	V	V	L	N
LC 75	CV	P	V	V	L	N
UK661	VV	A	I	I	I	S
DV86	VV	A	I	I	I	S
Edgar	CV	P	I	V	L	N
F52/70	CV	P	I	V	L	N
Del E	AV	T	V	V	L	N
GLS	AV	T	V	V	L	N

VV, very virulent; CV, classical virulent; and AV, antigenic variant

**Fig. 4:**
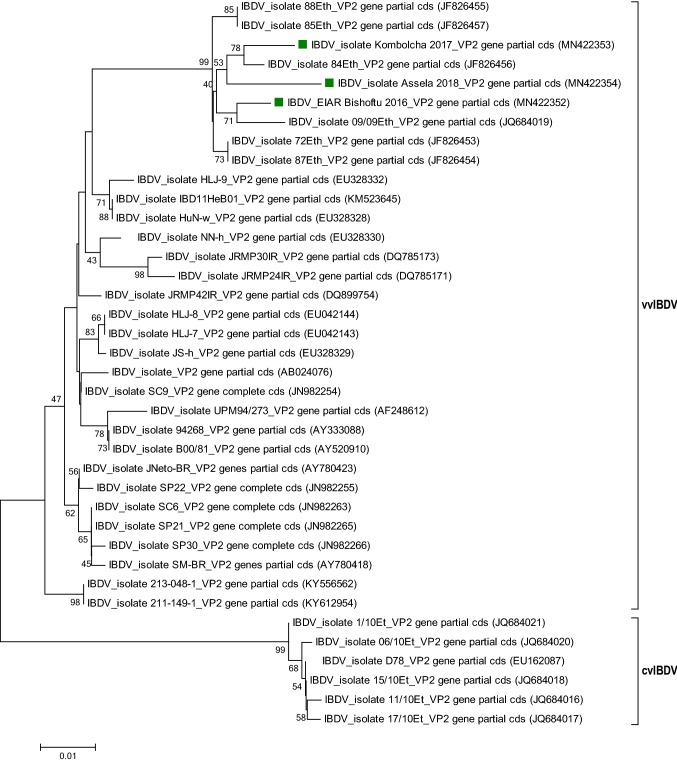
Phylogenetic tree based on a 750-bp region of the capsid protein 2 (VP2) gene of IBDV. The analysis included 38 nucleotide sequences: three from the present study and 35 from the GenBank database. The tree was constructed using the neighbor-joining algorithm in MEGA7, with 1000 bootstrap replicates. The IBDV isolates from this study clustered with very virulent IBDV isolates. The bootstrap values are displayed at the branch points. The three isolates sequenced in this study are indicated by a green square

Phylogenetic analysis of 38 IBD viruses based on the nucleotide sequences of the hypervariable region of the VP2 gene of IBDV field isolates, classical/attenuated vaccine strains, and reference sequences of classical and very virulent strains retrieved from the GenBank database showed that the isolates obtained in this study clustered with very virulent IBDV strains, together with previously characterized IBDV isolates from Ethiopia (Fig. 4).

## Discussion

In Ethiopia, chicken production is an important economic activity, serving the community as a source of cash and providing nutritional and socio-cultural services [7, 8]. The Ethiopian government has made chicken production a priority sector for achieving food security. The goal is to raise the number of small-scale and commercial poultry farms [24]. To achieve this goal, control of poultry diseases such as IBD is of vital importance. Understanding its epidemiology is important for optimal control using vaccines, and it is necessary to catalogue the strains circulating in the country. Based on the criteria used to identify very virulent IBDV (vvIBDV), mortality rates, nucleotide and amino acid sequences at key positions, and their phylogenetic position, the field strains isolated in this study are genotypically very virulent, which can break the high and normally protective levels of antibody. Very virulent IBDV strains have been identified in outbreaks inflicting up to 80% mortality on chicken flocks, resulting in considerable losses. Similar observations were reported previously by Jenberie et al. in 2013 [12], Jenberie et al. in 2014 [13], and Mekuriaw et al. in 2017 [18], when vvIBDV caused several outbreaks in Ethiopia. Taken together, the results should be taken as an alarm for the livestock service authorities. The veterinary service should reconsider the effectiveness of the vaccines used for control of IBD in their intervention programs.

The current isolates of IBDV have very similar VP2 sequences and appear to be closely related to previously reported Ethiopian isolates. The deduced amino acid sequence of the immunodominant epitope of VP2 from IBDV isolates from this study is identical to that of the European reference strains UK661 and DV86, except at position 222. Since the VP2 variable domain is subject to mutation, comparison of this region among various strains of IBDV provides better evolutionary information than comparison of other regions. Consistent with our observation, a previous study revealed that Asian and African vvIBDV strains belong to the common and very virulent European UK661 lineage [27]. This suggests a possible introduction of the virus into Ethiopia from Europe, as poultry breeding stock are mainly imported from central European countries. This is further supported by the fact that the first confirmed case of IBD was reported on a commercial farm where a live attenuated vaccine imported from the Netherlands had been used. Evidence from the literature suggests that vvIBDV strain DV86, which was first detected in the Netherlands in 1986, spread from there to various parts of the world [5].

The commercially available IBD vaccines produced and used in Ethiopia are based on strains with intermediate virulence. Vaccines prepared from classical strains have been shown to provide protection against vvIBDV strains [23, 28]. However, these vaccines are affected by maternally derived antibodies, suggesting a possible spillover of vvIBD viruses in the vaccinated flock. It has been shown that vaccines prepared from cvIBDV strains provide partial protection against vvIBDV strains, ultimately allowing infection of vaccinated chickens, which then excrete the virulent viruses. This is considered one of the reasons for the appearance of mutant vaccine strains causing outbreaks in vaccinated flocks [2]. The adaptation of escape mutant vaccine strains in regions using vaccines prepared from cvIBDV strains alongside indigenous field strains has been reported [1, 11]. Moreover, the rampant use of live vaccines, especially imported ones without genotype matching, has resulted in genetic diversification of circulating viruses through assortment, as has been observed in Poland [5]. Further studies are needed to evaluate the efficacy of commercial vaccines for protecting chickens against virulent field strains, but we suggest that effective protection against vvIBDV might be achieved if vaccines are prepared from autogenous strains causing outbreaks.

Infectious bursal disease continues to be a problem for poultry farmers. Since IBD is primarily controlled using vaccination, the failure of vaccines to elicit adequate immunity in young chicks must be considered. Several factors can cause vaccination programs to fail, including the lack of complete cross-protection among all strains. As IBDV continues to evolve through genetic mutation, continual surveillance for antigenically unique viruses is needed. Field viruses must be screened rapidly for unique mutations that are known to cause antigenic drift, resulting in the ability of IBDV to circumvent immunity generated using current classic vaccines. Analysis of the isolates IBDV_EIAR, IBDV_MB267, and IBDV_MB38 and other reference and vaccinal strains of IBDV suggested that the present field isolates originated from a common ancestor. Their partial VP2 sequences are identical to those of other Ethiopian and European vvIBDV strains, but they differ from those of the vaccine strains D78 and LC75. The results of the phylogenetic analysis in this study are consistent with those of Kasanga et al. in 2007 [14], Mohammed et al. in 2014 [19], El-Bagoury et al. in 2015 [6], and Ndashe et al. in 2016 [21], which demonstrate the continuous evolution and mutation of the VP2 gene of IBDV. This can affect the antigenicity and pathogenicity of the virus [20, 28]. Mutations at amino acid positions 206 to 350 of VP2 have resulted in changes in virulence and have also affected the antigenicity of this protein and the effects of neutralizing antibodies [5]. Continued vigilance in monitoring field viruses and understanding the natural history of the disease in needed.

In conclusion, this study revealed that vvIBDV strains are circulating in Ethiopia, inflicting considerable losses on poultry farmers and distribution centers. We have identified amino acid differences between the local strains and vaccine strains. Thus, preparation of vaccines from local strains to control IBD in areas where outbreaks might not be controlled by commercially available vaccines is suggested. Investigation of the neutralizing effects of monoclonal antibodies against field strains is planned. Further molecular characterization of IBDV isolates from outbreak areas targeting the different production systems should be conducted regularly to obtain pathogenic genotyping data for the circulating viruses.
